# Book review of the adaptable mind: What neuroplasticity and neural reuse tells us about language and cognition

**DOI:** 10.3389/fnins.2022.978196

**Published:** 2022-11-10

**Authors:** Xueyao Pan, Xi Li

**Affiliations:** ^1^English College, Center for Language and Brain Sciences, Sichuan International Studies University, Shapingba, China; ^2^Foreign Language College, Chengdu Normal University, Wenjiang, China

**Keywords:** brain, adaptable mind, neuroplasticity, neural reuse, language

## Abstract

From the works of Noam Chomsky to Jerry Fodor, the modularity of mind has been deeply rooted in cognitive science. From the “modular” perspective, cognition consists of functionally and anatomically isolable subsystems. Therefore, it is reasonable to assume a language module or a hardwired language organ that is functionally and anatomically different from the other cognitive modules (e.g., vision, olfaction, motor). However, evidence from neuroscience casts doubt on this modular hypothesis. It has been shown that many brain regions are likely to be reused and recycled by various neural communities in order to serve various cognitive functions. If this is the case, language and other cognitive faculties should not be considered modules, since they cannot be realized in special-purpose, special-structure regions. Therefore, it is time to change our understanding of our brains.

In this book, John Zerilli revisits the notion of modularity to accommodate recent evidence of neural reuse, which may provide a clearer and far more realistic picture of how the brain works. Bringing together cutting-edge research from neuroscience, cognitive science, linguistics, and philosophy, this book makes a valuable contribution to investigating brain modularity in general and language modularity in particular, with the potential to inspire further research in language and cognition.

The book consists of nine chapters, and can be roughly divided into five parts. Part I (Chapters 2 and 3) provides an overview of neuroplasticity and neural reuse. Part II (Chapters 4–6) investigates some of the controversial issues surrounding modularity and unveils the implications of neuroplasticity and neural reuse on modularity. Part III (Chapter 7) reconsiders the Language Module from the perspective of neural reuse and neuroplasticity. Part IV (Chapter 8) casts doubt on the claim that psychological states are multiply realized.

Chapter 1 introduces a much-debated question: that is, whether the mind is a modular system. For this question, there are two mutually exclusive views: modularism and holism. Modularism holds that the mind is a complex system, composed of different subsystems serving different functions. On the contrary, holism suggests that the mind is impenetrable, and every part is equipotential. A module can be characterized as specialized, autonomous, functional, dissociable, or innate.

Language has long been regarded as a module, as suggested by Noam Chomsky. However, Zerilli argues that, though language may be subserved by module-like entities, it cannot be regarded as a real module due to the lack of functional specialization. The final part of this chapter outlines the structure of the rest of the book.

Chapter 2 reviews neuroplasticity. Our brains and nervous systems are intrinsically characterized by plasticity. Neuroplasticity refers to the “capacity of the nervous system to modify its organization in response to experience.” Synaptic plasticity is the most well-known type of neuroplasticity. It may be the base of cortical map plasticity (e.g., language cross-lateralization after injury) and memory consolidation (e.g., through hippocampal long-term potentiation). Cortical map plasticity includes intramodal and crossmodal plasticity. Cortical intramodal reorganization occurs when a given cortical area is deprived of its normal afferent input. This area can then respond to the input with the same modality that was formerly captured by the adjacent areas. Crossmodal reorganization happens when a deprived cortical area begins to respond to input from another modality that would usually be processed by a different cortical area. Crossmodal plasticity actually reflects the metamodal or supramodal nature of the brain, which in turn indicates that no area is domain-specific or modality-specific. In this sense, the view of the mind as strictly modular should be rejected.

Chapter 3 provides a brief survey of neural reuse. Due to the brain's metamodal organization, some low-level neural circuits can be reused to support high-level cognitive processing while keeping their original functions. Neural reuse indicates that there is no such thing as a language module. Even the traditional language-specific areas, such as Broca's region, may play a role outside the domain of language because this area includes numerous subregions or functional units that can be reused. Anderson holds that neural reuse is the result of evolution. His Massive Redeployment Hypothesis posits that a brain region can support numerous cognitive functions. The brain regions that evolved earlier are more likely to be reused, while the more evolved functions may involve more distributed areas.

In Chapter 4, the notion of modules is recalibrated to accommodate evidence from neuroscience, and a “soft” version of modularity is proposed, with functional dissociability as the *sine qua non*. The investigation of modularity can be carried out following two approaches: the functional and the anatomical. The former holds that human cognition consists of various functionally independent cognitive modules. A module, as suggested by Jerry Fodor, is “a domain-specific, innately specified, and informationally encapsulated system.” The latter further indicates that each cognitive module resides in some specific portion of the brain. In other words, functional modularity is somewhat “soft,” emphasizing functional dissociability, while anatomical modularity is relatively “hard,” focusing on both functional dissociability and anatomical dissociability. According to graph theory and network neuroscience, a softer version of modularity can be developed. A module can be seen as a community of functionally interconnected and coactivated codes. Thus, the functional specificity of a module is due to the global structure of the community, rather than the individual nodes themselves.

Chapter 5 aims to unveil the neural substrates of modularity, with the evidence of neural reuse taken into account. One candidate that meets the standard of a “soft” version of modularity is cortical columns, which are small, stable, reusable nodes that can be found in various distributed networks and can be involved in various cognitive domains. However, some researchers have noticed that the network context can influence local functions. Minimodules, such as the cortical columns, may lack a precise degree of specialization since they may be recruited by multiple diverse neural communities. The more partnerships a given minimodule enters into, the more abstract its contribution becomes, and the more generic it will ultimately be. In this sense, it cannot be considered a genuine module. Actually, what matters is not the specific substrates, but the scale of specificity for brain regions that support different degrees of functional specificity.

Chapter 6 argues that neuroplasticity and neural reuse are not unconstrained. Our brain is innately organized, as cortical development seems to be scheduled and activity-independent (e.g., prenatal cell differentiation). Additionally, brain regions seem to be robust when encountering learning, injury, and sensory deprivation. The relative “innateness” of the brain does not suggest that brain regions are real modules. Instead, it indicates that the brain is not “open-endedly malleable.” In some cases, some instances of the metamodal brain may be misinterpreted as neuroplasticity. The metamodal nature suggests that brain regions may be structured to process multimodal inputs. Therefore, when the best fit input is unavailable, one region can easily handle another kind of input without radical (structural) change. Even if a fundamental change occurs, it may be restricted to a certain cortical site. Reconfiguration occurs only within the site that shares structural features with the impaired site. Therefore, if the language circuits in the left hemisphere have been impaired, for example, only the counterpart site of the right hemisphere can shoulder the responsibility.

Chapter 7 reconsiders the issue of language modules and aims to resolve the conflicts between linguistic modularization and neural reuse under the Redundancy Model. Language is indeed supported by defined sets of neural circuits, but whether these circuits are specific is the subject of debate. If there is a language module, it may be a composite structure that consists of several sub-modules supporting complex functions. The neurological approach to the modularization of language suggests that, as long as one constituent node or part is functionally specialized, the whole language network can be seen as a functionally specialized module. However, there seems to be no such specialized elementary linguistic unit. For example, Broca's area, an important part of the so-called “language module,” can also be engaged by various action-related tasks. On the contrary, the psychological approach indicates that the uniqueness of the interconnections between nodes determines the specialization of language. Whether the constituent nodes are language-specific or domain-general may not matter. However, there seem to be no hardwired interconnections on different occasions. From an evolutionary perspective, a language module is not necessary. As a recently evolved function, language is more likely to reuse and adapt existing resources in the brain than to evolve new systems from scratch. In addition, language and cultural environments are subject to changes, and unstable environments are not suitable for the development of language-specific systems. The language module is also unnecessary. Although language seems to be acquired effortlessly, regardless of the poverty of stimulus, this may not necessitate an innate language module. Language is culturally shaped to be learned easily, and the more cumbersome or exotic elements of languages tend to be discarded over time. Moreover, the processing dispositions of the brain regions involved, as shaped byevolutionary pressures, also contribute to the learnability of language. However, caution should also be applied to the use of the neural reuse theory. Apart from neural reuse, our brain is also characterized by neural redundancy; i.e., neighboring cortical areas have similar basic structures and response properties. Therefore, in the same cortical zones, we have several copies of the “module” rather than only one “module” for a certain cognitive function. In other words, neural reuse suggests that the same neural tokens are recruited to accomplish linguistic and other tasks, while neural redundancy indicates that the same neural types are engaged in different tasks. Perhaps due to the redundant nature, the dissociations between linguistic and nonlinguistic capacities can be observed, though the evidence of dissociation alone cannot fully prove the functional specificity.

Chapter 8 attempts to show that the study of psychology should not neglect the discoveries of neuroscience. Some proponents of traditional psychological faculties argue that, just as a computer's hardware has no direct relationship with the software installed on it, neuroscience alone can tell us nothing about the nature of higher-level cognitive systems, because the psychological processes are multiply realized. The Multiple Realization (MR) Hypothesis insists that a certain cognitive state can be realized by many neutrally distinct substrates. The many-to-one mapping from neural states to mental states further indicates that the evidence from the field of neuroscience may provide less implication for the understanding of psychological processes. However, the author holds that the primary empirical arguments for MR, such as neuroplasticity and convergent evolution, may be open to doubt. Therefore, psychology and neuroscience may not be mutually exclusive.

Chapter 9 concludes the book. The functionally and anatomically specific modules, in the traditional sense, are exiguous when neuroscientific evidence is taken into account. Therefore, the notion of modularity should be revised. By combining the discoveries of psychology and neuroscience, we may gain a better understanding of how the brain/mind works.

In summary, this book offers an insightful account of brain modularity, making clear the connections between language, other cognitive systems, and the brain. This work will generate fresh insights into linguistic ability for the following reasons:

First, it encourages us to reflect on the traditional view of language. Classical cognition holds that the language system is an independent module in the brain, sandwiched by the modules for perception and action (Hurley, 2001). Language processing is the symbol manipulation in the language module, which cannot be influenced by other domains. A great deal of effort has been put into exploring the distinctive brain regions supporting language. Some candidates have been proposed, such as Broca's area, Wernicke's area, and the angular gyrus. However, why these areas are specific to language, and how they can contribute to language processing, is still being questioned. Besides, as “language is not one thing but many things,” it seemed unlikely that some areas alone can contribute to such a complicated function. Therefore, the module view of language should be updated; a “global workspace view” may be more convincing.

Second, this book attempts to provide further theoretical and empirical support for embodied or grounded language. According to the grounded cognition approach, any high-level cognitive system, including the language system, is not self-sufficient but depends on the lower-level perceptual systems. Additionally, higher-level cognition and lower-level perception share the same neural substrates. Language also exploits the neural regions involved in perception and action (e.g., the sensory-motor areas). The neural exploitation hypothesis of language (Gallese and Lakoff, 2005), the action-based language theory (Glenberg and Gallese, 2012), and the action-perception circuits for language (Pulvermüller, 2018) all emphasize the role of the sensory and motor systems in supporting language. However, in contrast to the radical grounded theory, this book distinguishes the neural type from the neural token involved in different cognitive functions. Due to neural redundancy, the same types, but not the same token, of neural substrates are recruited in language and other cognitive tasks. In other words, the language system is not identical to other systems, which seems to corroborate the idea of a weak version of embodied cognition.

Although this book does not provide a clear-cut answer for the mystery of language, its refuting of “language modularity” may be less simplistic. The author investigates the language module on the base of Chomsky's and Fodor's work. However, the language module proposed by Chomsky was just a “functional module”, which lacks specific neural substrates. Moreover, the language area mentioned by the author is limited, whereas language processing involves a large-scale network, such as the orbital frontal-temporal occipital network, the opercular/triangular middle frontal-subcortical module network, and the middle temporal lobe (Fang et al., 2015). Broca's area is only the tip of the iceberg. Whether these are functionally and anatomically specialized for language is not clear. Nevertheless, this book will benefit researchers who are interested in the relationship between language, mind, and brain. It may also have important implications for language rehabilitation, language learning, and artificial intelligence.

## Author contributions

All authors listed have made a substantial, direct, and intellectual contribution to the work and approved it for publication.

## Funding

This work was supported by the National Education Science-13th Five Year Plan 2018 Youth Project Research and Practice of College Oral English Teaching Based on Mobile Terminal (ECA180464).

## Conflict of interest

The authors declare that the research was conducted in the absence of any commercial or financial relationships that could be construed as a potential conflict of interest.

## Publisher's note

All claims expressed in this article are solely those of the authors and do not necessarily represent those of their affiliated organizations, or those of the publisher, the editors and the reviewers. Any product that may be evaluated in this article, or claim that may be made by its manufacturer, is not guaranteed or endorsed by the publisher.
